# Damaged Goods

**Published:** 1912-02

**Authors:** 


### "DAMAGED GOODS."

**Syphilis in Relation to Marriage**

This is the title of one of "Three Plays," by Brieux (see Book Notices). It deals with the effects of syphilis in relation to marriage. It is an object lesson which, if it could be popularized and produced in our theaters would do an immense amount of good. The mock-modesty of society, thus far, has tabooed it, and so far as I am aware it has not been played before the general public, even in Paris or London;—but has been repeatedly played before the "Stage Club" in London.

A young man is engaged to be married to an heiress and is very anxious to consummate the engagement. He consults an eminent specialist who, after a blood examination, informs him that he has syphilis, and warns him most earnestly against marrying, setting forth in eloquent language the terrible results that are sure to follow. The doctor insists that he should not marry in less time than four years, during which time he should be treated and cured. The young man disregards the advice and is married. * * * A lovely little daughter of two years, the idol and heiress presumptive of a doting and very wealthy grandmother, shows signs of failing health and is sent to the country. A peasant woman—a mother—is employed as wet nurse. The family physician finds syphilitic patches in the child's mouth, and warns the grandmother that the nurse would contract the disease and must be discharged, having babies of her own. The scene between the woman and the grandame and that which follows between them and the mother of the child is dramatic, nay, as tragic as anything in Aeschylus, Euripides or Shakespeare, although as Shaw says, "No one gets killed or married." The grandmother wants to keep the nurse, and offers her large sums to stay. But the doctor insists that she be discharged, and the nurse claims the promised pay and a quarrel ensues. Grandmother takes nurse by the arm, saying, "Hold your tongue!" The now frantic nurse screams, "Let me go! Let me go! I know your brat is not going to live! I know it is rotten, through and through, because its father's got a beastly disease that he caught from some woman of the streets!"

The mother with two hoarse cries falls to the ground in a fit of nervous sobbing. Her husband rushing to her exclaims, "My God!" The wife eludes him, and with disgust, hatred and horror depicted in her face, shrieks, "Don't touch me! don't touch me!" She then takes her child and goes to her father.

The great specialist's plea with the young man is a most eloquent and enlightening lecture, alone worth many times the cost of the book; and the introductory by Bernard Shaw is a gem.

Another of the plays is entitled, "Maternity," and is a striking illustration of the folly of encouraging large families amongst the laboring class whose wages—*when they can get work*—barely suffice to support a family of two or three; a verification, in fact, of the dictum of Malthus as to population and food. (Malthus, 1798. *"Essay on the Principles of Population as it affects the future Improvement of Society."* "If population were permitted to increase at its normal rate, it would soon overtake the means of subsistence.") In densely populated countries we now have famine. In large cities the excess of poor children, thrown on charity or the street, become criminals or paupers. Small families is not race suicide; quite the reverse. The indiscriminate production of people, mostly defective, is race suicide, and it is truly astonishing that the great and wise apostle of big families—Mr. Roosevelt—should not know it. More on this subject in next issue.

---

SHOULD THE "CANTEEN" BE RESTORED?—The consensus of medical opinion is that the physiological action of alcohol is that of an acrid, devitalizing, narcotic poison upon every tissue and fluid of the human body, spending its poisonous influence, chiefly upon the brain and nervous system, and upon the liver and kidneys; that this action is certain, constant and uniform, and *pari passu* with the quantity consumed, whether in the form of "light beer," "high-balls," "cocktails," "mint julep," "long toddies" or whether taken "straight."

In the light of the foregoing facts—for facts they are, beyond gainsaying—it is almost beyond belief that *two hundred and seventy-five* of the leading medical men and teachers of the United States could be found lending their influence toward restoring the "canteen" to the army; every one of whom, who teaches medicine in any of its branches, daily teach their students of the baneful influence of alcohol on the human body, whilst all of them, as practicing physicians, are constantly and assiduously endeavoring to counteract the mischief which this fell destroyer has worked on thousands of their patients.

The plea on which these distinguished members of the medical profession base their appeal to the Senate and House of Representatives for restoring the "canteen" that it would be in the interest of temperance; of military order and efficiency, and of the health and happiness of our soldiers and of their future wives and children," is the acme of puerile reasoning, if it be reasoning at all. The idea that the *"esprit de corps"* of the soldiers of the U. S. army, their health and happiness, and the health and happiness of their future wives and children could be jeopardized in the least because they can not buy beer at the army "canteen," is not only preposterous, but is slanderous as well, and the army, to a man, "horse, foot and dragoon," should repel it as slanderous.

It is also well known that alcohol, when not taken to drunkenness—in about the supposed quantity that a "soldier in good standing" would be permitted to buy at the "canteen"—excites the sexual passions of both men and women. How then could it be expected that restoration of the "canteen"—where the soldiers are daily permitted to inflame these passions, those "in good standing," to the extent of one-fifth of their pay, *on credit*—would prevent them from resorting to "the dens of dissipation and disease just beyond the jurisdiction of the commanding officer *where they are further supplied with impure and often drugged whisky, robbed of their hard earned pay, and often, if not indeed generally, contract disease"?* [Writer's italics.]

Would not abolition of these "dens of dissipation and disease" be a surer, a more rational and a more moral way of protecting the soldier from these abuses and outrages? The dens are there because they pay the government—directly or indirectly—for that privilege; and if after having been encouraged by the government to excite his sexual desires and desire for drink with "light beer" at the army "canteen," he resorts to them in order to satisfy his cravings, then the government becomes *particeps criminis* in the soldier's downfall, in the loss of his money, and in the loss of his health; for it is the duty of the government to protect the lives, health and happiness of its citizens, and to abolish whatever tends to the destruction of either.

During all the years of the use of alcohol as a beverage, in whatever form, from whatever source, "canteen" or barroom, there is no positive proof of its having been of benefit to a single human being; whilst on the other hand, our repleted graveyards, overflowing insane asylums, crowded jails, hospitals, poorhouses, penitentiaries and dispensaries attest to its awful ravages, not to write of ruined homes, blasted hopes and squandered fortunes; the oceans of tears it has wrung from those whom it has widowed and orphaned, and from girls whom it has debauched and robbed of their virtue.

With that portion of the petition which embraces the recommendation of the Surgeon General to form "clubs, where the soldiers can find amusement and recreation; * * * temperance associations; * * * instruction by lectures and * * * by informal advice * * * as to the nature of venereal diseases; * * * their prevalence among prostitutes, and the danger to themselves and their posterity," I am in hearty accord; but in none of this does it appear that "light beer" can be necessary. What would be thought of a minister of the gospel who would recommend that his congregation be served such refreshments in the interest of temperance and virtue?

It is asserted in the "petition" also that since abolition of the "canteen," in 1901, desertions from the army have increased. This may be true; but it does not necessarily follow that they are due to that cause, for it is past believing that the patriotism of our soldiers—the very best men, physically, that the nation affords—depends upon their ability to buy a glass or two of "light beer" at the army "canteen"; but should it be true that some have deserted for that reason, the sooner the army is rid of such material the better, for men of that sort could never be depended on "when none but Americans must stand guard tonight." They never make soldiers. On the contrary, they form the horde of prowling stragglers of an army on the march; the rear ranks when a battle is to be fought; the front line when pension time comes around; and no means should be taken to retain them; their desertion should rather be regarded in the light of a desirable riddance than as a loss. Some writers in recent magazine articles have declared that "Man's inhumanity to man" has caused more desertions than all other causes combined. The futility then of trying to prevent desertions—if the charge be true—by restoring the "canteen," is too apparent to merit additional comment.

By all means let the soldiers have their diversions and recreations in the way of club rooms, billiard halls, pianos, gymnasiums, temperance lectures, schoolrooms, moving pictures, lectures on health, informal advice as to the dangers of contracting venereal diseases from prostitutes, the danger that these diseases entail upon them and upon their posterity; let them have their dancing halls and histrionic entertainments; yes, all these and more, supplied by the government, "without money and without price"; but for the sake of the soldier, for temperance sake, for morality and for the sake of the soldiers' wives and children, present and future, let no "light beer" or other beverage that can intoxicate enter into either of these for fear it might be a stumbling block to cause the downfall and eternal undoing of a single "soldier in good standing."

The recommendation of the Surgeon General that the soldiers "should be taught that sexual intercourse is not necessary to good health and the highest degree of mental and physical vigor," is timely and should be acted on, but why not write that they should be taught that neither sexual intercourse nor beer drinking is necessary to the attainment of this high degree of perfection? The one is natural to man, although not necessary to health and vigor; a manifestation of the *instinct* for racial *perpetuation;* the other is an exotic *vice* which tends to all manner of excesses and abuses and to racial *extinction.*

What would the petitioners have thought of the Surgeon General if *"in the interest of temperance and of virtue,"* that functionary had seen fit to recommend establishing a salon in connection with the "canteen," where the "soldiers in good standing" might find aseptic sexual refreshments, under proper regulations and official surveillance? Such an idea is extremely repugnant to even the crudest conceptions of morality, and the writer craves the pardon of his readers for having used it as an illustration; but he is free to state that in his opinion its adoption would do more to lessen the number of venereal diseases among our soldiers than would restoration of the "canteen." The statistics cited in the petition indicate that venereal diseases are much less prevalent among the nations who subject prostitutes to strict police regulations and to rigid inspection than they are among the nations who make no such provision for this class of their citizens.

R. H. L. Bibb, M. D.

(Worse and Worse.—The daily papers announce that the wife of Major General Fred Grant and twenty-four hundred other ladies, mostly wives of army officers, have petitioned Congress to *restore the canteen.*—Daniel.)

THE MENINGITIS EPIDEMIC in Texas is creating widespread alarm, and well it may, considering that the medical profession frankly admit that they know very little about it, either as to how, when and where it starts, its cause or mode of spreading or its treatment. The fact that it seems to skip over large areas of country—from Clarksville, in the extreme northeast, to Houston, San Antonio, Waco and other interior towns, seems to indicate that the disease is contagious; but the profession is widely divided on this point, all admitting, however, that it is infectious, and all assume that it is caused by a germ they call meningo-coccus. As a result of this panic and want of knowledge of the cause of the disease and its propagation quarantine is resorted to, clean-up crusades, inspection of all premises and fines for insanitary conditions, fumigation, the closure of schools, prohibition of assemblages, etc., are resorted to, and we expect to see a recurrence of shotgun methods as in the days of our ignorance about yellow fever. We used to do all those things and we see now how absurd they were. No one knows what, if any, relation neglect of sanitation bears to the meningitis, nor whether it is carried in "fomites"—hence whether or not fumigation does any good. When Sternberg, Surgeon General, U. S. A., just about the time the mosquito doctrine was fighting for acceptance, published a statement that filth had nothing to do with the origin or spread of yellow fever, Reed, Carroll, Agramante and Lazear having just demonstrated it, and shown that black vomit or feces of a yellow fever patient was not dangerous, a howl was raised by Southern physicians because it struck at their cherished belief in *fomites* and that an epidemic of yellow fever would light up if goods or persons from an infected place were allowed to enter a town which was not clean; and despite all the proofs and demonstrations of the absurdity of quarantine and disinfection both were kept up in Texas until—almost now—as to yellow fever. It is related that as late as 1900 the then State Health Officer quarantined and fumigated a flat car of railroad iron, and all mails were opened and fumigated, officially, even by the Marine Hospital surgeons.

The day will surely come when science having unlocked the secret that now so alarms the people we will laugh at the frantic guesses—and the frantic efforts to guard against an unknown enemy—a danger in the dark—the more terrible for that reason, just as we now laugh at the same things we did in yellow fever days. A serum said to have been first made by Jager* in 1895 is about the only treatment relied upon. It is injected into the meningeal membrane of the spine, and reports indicate that it has reduced the mortality to some extent.

*The *diplococcus intercellularis meningitidis* was discovered in 1887 by Weichselbaum, and proved by Jager in 1895 to be the cause of cerebro-spinal meningitis, and found, during the disease, in the cerebro-spinal fluid.

